# Decision-Making under Risk: Conditions Affecting the Risk Preferences of Politicians in Digitalization

**DOI:** 10.3390/ijerph19053036

**Published:** 2022-03-04

**Authors:** Jean Roisse Rodrigues Ferreira

**Affiliations:** Department of Media and Social Sciences, University of Stavanger, P.O. Box 8600, 4036 Stavanger, Norway; jean.roisse@uis.no

**Keywords:** digitalization, health, data privacy, political decision-making, framing, prospect theory, multiple dimensions

## Abstract

Public officials are constantly facing decisions under risk, particularly in digitalization policies, the consequences of which are hard to predict given their multiple dimensional nature. Since scholarly research has not yet addressed this phenomenon, we do not know what influences the risk preferences of politicians in digitalization policies. Prospect theory—widely used to explain political decisions—can help us describe politicians’ potential risk references and the conditions affecting their decisions. Accordingly, this paper aims to answer the following question: what are the conditions affecting the risk preferences of politicians in digitalization policies? I address this question by employing two important assumptions of prospect theory: the value function and the probability weighting function. Particularly, I discuss the effects of loss/gain frames and probability weighting on the risk preferences of politicians in digitalization with outcomes in multiple dimensions (e.g., data privacy and economy). I argue that whether an outcome is perceived as a gain or as a loss depends on how the situation is framed and how the probabilities are weighted. I conclude with a brief discussion of how prospect theory can leverage our understanding of political decisions in highly complex policy environments.

## 1. Introduction

The emergence of digital technologies is pushing public officials at the national and subnational levels to increasingly deal with decisions involving digitalization—broadly defined as the use of information and communication technologies (ICTs) to alter or adopt processes [1]—of key areas such as healthcare. This has created a new and uncertain landscape for political decision-making. This is largely because it is difficult to predict whether the outcomes of digitalization are positive or negative [2] given its multidimensional nature: while some of the outcomes in digitalization are associated with the economic dimension, particularly the impacts on jobs [3,4], others fall within the cybersecurity dimension, especially data privacy [5].

This high level of uncertainty creates a high-risk decision-making environment for politicians. McDermott [6] argues that it is the uncertainty of outcomes that makes decisions risky, which is a major aspect to consider in political decisions, as they are by nature highly uncertain. Nevertheless, scholarly research has yet to catch up with this new phenomenon in political decision-making. For the most part, research on digitalization has been concerned with digital technology for citizenship and political participation [7], information technologies (e-government) adoptions [8,9], public sector efficiency [10], smart government [11,12], and public administration and big data [13].

One theory that offers a systematic way to examine decision-making under risk and uncertainty and that has been employed in recent years by scholars to describe and measure decisions and biases among elected politicians [14,15,16,17,18] is prospect theory [19]. Put simply, prospect theory predicts that people are risk-averse under gain frames and risk-seeking under loss frames, which is captured by the value function, and that they tend to weight probabilities nonlinearly, which is predicted by the probability weighting function.

Accordingly, this paper seeks to contribute to the literature by employing prospect theory to describe the conditions affecting the risk preferences of politicians in digitalization with outcomes in multiple dimensions. More specifically, it aims to answer the following question: what are the conditions affecting the risk preferences of politicians in digitalization policies? I address this question by employing a theoretical discussion of how loss/gain frames and outcome probabilities affect the risk preferences of politicians in digitalization. Particularly, I argue that the reference point, which determines whether an outcome is perceived as a gain or as a loss, depends on how the situation is framed. Then, I indicate that the probability weighting function needs to be considered, as preferences can be reversed depending on the probability of success or failure of the policy. Overall, the contribution of this paper lies in how prospect theory can be used to describe the risk preferences of politicians when outcomes are multidimensional.

The remainder of this paper proceeds as follows. First, I briefly discuss the literature on prospect theory and lay down an analytical framework. Second, I employ the analytical framework to investigate the risk preferences of politicians in digitalization involving the economic and data privacy dimensions. Third, I conclude with considerations and implications.

## 2. Prospect Theory: Loss/Gain Frames and Probability Weighting

**Figure 1:**
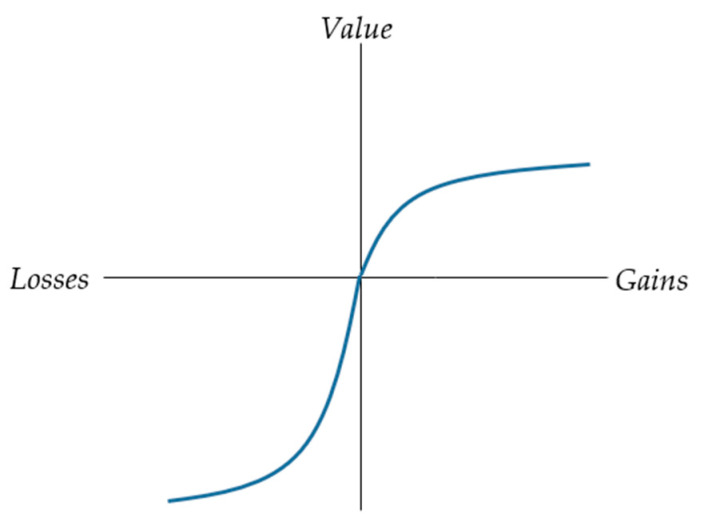
The S-shape value function captures risk-aversion for gains but risk-seeking for losses.

Prospect theory [19,20]—considered one of the most important descriptive models for risk and ambiguity [21,22]—confronts expected utility theory by asserting that individuals make decisions based on deviations from a reference point rather than on total wealth. People tend to be risk-averse under the positive frame and risk-seeking under the negative frame, which is equivalent to a two-fold pattern of risk attitudes. This pattern of choices indicates the existence of a framing effect, predicted by the S shape of the value function (Figure 1): concave for gains indicating risk aversion and convex for losses indicating risk-seeking [23].

This conceptual insight is summarized by the reference dependence [24] (p. 1039), which argues that people deal with gains and losses differently: they overweight losses in relation to equivalent gains, which is captured by loss aversion [19,25]. This is further supported by the status quo bias [26], meaning that people usually prefer to remain at the status quo due to their tendency to overweight the costs relative to the benefits of moving away from the status quo [27]. In other words, the status quo is taken as the reference point, and people tend to perceive any changes away from it as costs. Risk preference, therefore, depends on how gains and losses are framed, and the reference point plays an important role in defining these domains. Accordingly, “the identification, or framing, of the reference point” strongly contributes to people’s risk preferences. Changing the frame from gains to losses can reverse the risk preference [27] (p. 90).

In many cases, the framing of a choice is mostly predefined by the situation. McDermott [18] argues that although prospect theory starts at the individual level of analysis, it also depends on situational factors as important determinants of decisions. She refers to the situation as the circumstances of the moment. Context, in this case, provides a larger temporal sense to a decision, which comprises the history of the event, the actors involved, and the trajectory (p. 300). Thus, risk propensity should also account for the situation in terms of losses (costs) and gains (opportunities) and not only for the individual characteristics [6] (p. 02). Using the situation to determine the domain is not easy, given the challenge of translating wide notions of a situation into operational domains. One way to overcome this is by determining a particular realm, or domain, which politicians consider most. Once this has been established, it becomes easier to assess the risk preferences of politicians [18].

For political actors, the status quo, the current situation, is usually used as the reference point to assess their domain: satisfaction with the status quo means they are in a domain of gain, while unsatisfaction with the status quo signals a domain of loss [17]. Situational factors such as domestic policies and economic conditions are also important benchmarks to determine the relevant reference points [17] (p. 05). For instance, economic instability tends to place politicians in a state of loss. This motivates political actors to embrace risky economic policies to recover costs and hence bring back economic stability [28]. The pre-crisis economic stability is therefore the desired status quo (i.e., the reference point) to which political actors want to return.

Mercer [17] notices that as policies are often incoherent and evolving with the situation, it is common to “find evidence of different reference points” (p. 06). This is the case in digitalization policies. Risk preferences in digitalization policies are affected by multitude of dimensions, which create different domains that influence political actors’ decisions (e.g., increased number of jobs (domain of loss) and lower data privacy security (domain of gain)).

**Figure 2:**
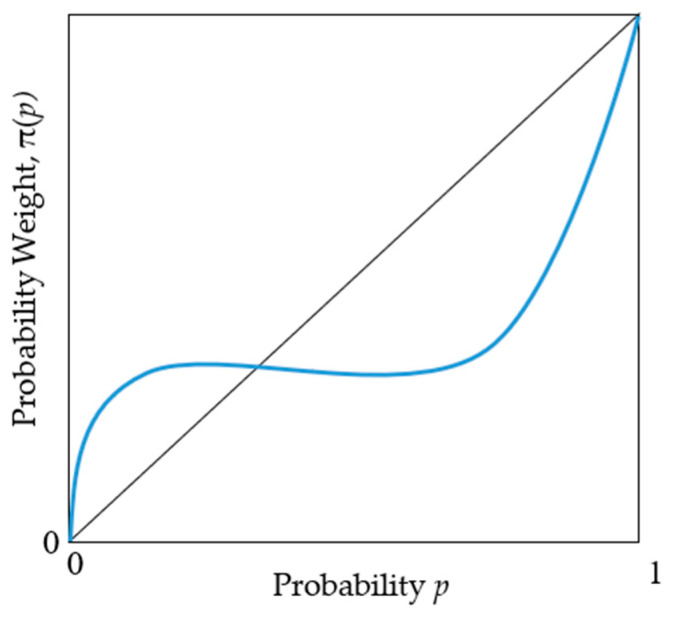
Probability weighting function: inverse-S-shaped, which is concave for low probability and convex for high probability.

There are two key aspects to be considered here. First, the framing of a policy is essential to identify the reference point, as the framing sets the basis to identify and describe the actors’ decision-making reference point and consequently their risk preference, given that positive framing leads to risk-aversion and negative framing to risk-seeking behavior. Second, risk preferences are determined not only by the value function but also by the probability weighting function, as individuals have “different attitudes toward probability of gains and losses” [29] (p. 55). Non-linear probability weighting is an integral part of descriptive theories of choice under risk, such as prospect theory, as they reflect objective errors in how individuals process information and deal with probabilities: they tend to overweight small probabilities but underweight the likelihood of large probabilities, with the second case being more salient than the first one [30]. This indicates that risk preferences are determined not solely by the shape of the value function but also by the shape of the probability weighting function (Figure 2) [31]. The combination of the value and probability weighting functions to describe individual risk preferences is characterized by the modified version of prospect theory, called cumulative prospect theory (CPT), developed by Tversky and Kahneman [20].

The two key propositions in CPT are that (1) individuals are risk-averse in gains and risk-seeking in losses, as reflected in the S-shape of the value function (i.e., concave for gains, convex for losses), and (2) they tend to overweight the likelihood of small probabilities (*p* > 0.30) and underweight the likelihood of large probabilities, reflected in an inverse-S-shape probability weighting function [22] (p. 176), [32]. The combination of both functions yields a four-fold pattern of risk attitudes: risk seeking for low probability of gains and large probability of losses, and risk aversion for small-probability losses and large-probability gains [33].

One important aspect when employing probability weighting to explore risk preferences is that individuals tend to overweight the end of distributions (i.e., unlikely extreme outcomes are overweighted). This happens “because people are limited in their ability to comprehend and evaluate extreme probabilities, highly unlikely events are either ignored or overweighted, and the difference between high probability and certainty is either neglected or exaggerated” [19] (pp. 282–283). As a result, changes in probabilities near 0 or 1 have asymmetrically large effects on the evaluation of choices [30]. This characteristic needs to be considered in political decisions, particularly in those involving multiple dimensions and unlike consequences, which are the case for choices in digitization. I will return to this when discussing the possible scenarios involving political decisions in digitalization with outcomes in the economy and the data privacy dimensions.

In essence, looking at political decisions through the lens of the four-fold pattern of risk attitudes helps to better access the risk preferences of politicians in what I call a “competition” of frames and probabilities, which ultimately leads to a tradeoff between accepting one dimension at the expense of another. This is particularly important in decisions involving digitalization. Digitalization can, for instance, have a positive impact in one area, such as the economy, by creating new job opportunities or leveraging industry efficiency, but it can also harm another dimension, such as data privacy, by compromising the privacy of citizens’ personal information. In essence, digitalization is unpredictable and multidimensional by nature. In what follows, I investigate how the gain and loss frames affect the risk preferences of politicians and the role played by probability weighting in digitalization policies with outcomes in the economic and the data privacy dimensions.

## 3. Risk Preferences and the Economic Dimension

The strong reliance on economic indices across scholarly work using prospect theory to examine how political actors make risk decisions [34,35,36,37,38,39] indicates that the economy is an important reference point used by political actors.

This is particularly important, as evidence shows that digitalization has significant impacts on the economy. For instance, approximately 47 percent of total US employment is under the risk of being automated in the near future [40]. Nevertheless, the consequences of digitalization in the economic dimension can very well be framed as beneficial to the job market. The assumptions made by studies such as Frey and Osborne’s have been greatly questioned [41,42]. The job market revolution brought by ICTs over the last decades has been responsible for creating new jobs based on tasks that are still difficult to perform by a machine [43]. For instance, Amazon has been responsible for over 135,000 new jobs in the U.S. alone in 2017 [44]. In France, the internet has abolished 500,000 jobs over a 15-year period while creating 1.2 million new jobs over the same period [45].

In fact, predicting the effects of automation on the economy, and more specifically on jobs, is not easy, as effects can vary over time. Cave and ÓhÉigeartaigh [46] argue that the short- and long-term effects of technology should not be seen as two separate trends, as decisions made now might constrain decisions in the future. They say that while experts remain divided on the issues, it is rather realistic to expect significant economic disruptions from AI automation in the long run despite the short-term benefits. For instance, Milakis and colleagues [47] discuss that while automated cars might offer many benefits, such as reduced travel and car ownership costs, they might also lead to critical negative consequences for the automotive industry with respect to job loss. The balance between the short- and long-term effects of technology generates a great deal of uncertainty, which increases the risk of decisions. Politicians must balance the short- and long-term effects of their decisions, and with something so uncertain as technology, it becomes even harder to decide whether to support a given digitalization policy that might create, on the one hand, short-term benefits such as enhanced industry efficiency but, on the other hand, increase unemployment.

That is why portraying the outcomes of digitalization as a catalyst for job destruction rather than for job creation is most likely to make politicians feel at a loss, leading to risk-seeking behavior to recover the costs and return to the status quo. Similarly, stressing how badly instead of how well the current economy is performing (e.g., 8% unemployment rate instead of 92% of the workforce employed) is also likely to throw politicians into the domain of losses, inducing risk-seeking behavior.

However, risk preferences are determined not only by the value function for the domains of gain and losses but also by the probability weighting function [20]. For instance, let us assume that a politician is in the domain of gain in the economy (e.g., 92% of the workforce employed), and the probability of various outcomes of a proposed digitalization policy in the healthcare sector are described as the following: an 80% chance of success of increasing the number of jobs in the healthcare sector and a 20% chance of having no effect. Since people become cost sensitive and less willing to move away from the reference point, or the status quo (i.e., 92% of the workforce employed, in this case), under the domain of gains, politicians would most likely underweight the high probability of success and overweight the low probability of failure, which reduces the attractiveness of the positive gamble and therefore increases the likelihood that they become risk-averse and vote against the policy [6] (p. 32).

In contrast, a domain of loss in the economy (e.g., increasing unemployment rates from 6% to 8%) would prompt risk-seeking behavior. Let us suppose that the outcomes of a digitalization policy in the healthcare sector are described as an 80% chance of policy failure (e.g., higher unemployment rates due to task automation in the healthcare sector) and a 20% chance of having no effect; that is, the policy has no impact on the unemployment rate. In such a scenario, the policy becomes more attractive for two reasons. First, the salience of the issue—unemployment—induced by negative framing leads to risk-seeking behavior captured by the value function (i.e., risk-averse for gains, risk-seeking for losses). Second, the overweighting of small probabilities and the underweighting of large probabilities also leads to risk-seeking by attenuating the aversion to negative gambles [6].

This behavior is captured by loss aversion, which suggests that “losses loom larger than gains” [19]. Since people tend to be very sensitive toward small losses but insensitive toward large losses that may result from large deviations from the status quo [48], politicians in a domain of loss would be resistant toward a policy with small deviations from the status quo, their reference point, and would become more inclined toward a policy with large deviations from the status quo. In both cases, the framing, influenced by how the outcomes are displayed, influences the risk behavior. In the first case, the 92% employment rate creates a situation in which the utility is perceived as positive. This is captured by the attribute framing, where the description of an event in a positive (e.g., employment) or negative (e.g., unemployment) way influences people’s risk preferences [For a review of the typologies of framing, see [49]. Since loss aversion indicates that “people are much more sensitive to losses—even small losses—than to gains of the same magnitude” [22] (p. 175), people prefer to stay at the status quo and not to risk the sure outcome over outcomes with probabilistic changes of occurrence [50].

In the second case, the reference point is reallocated below the expected outcome (from 6% to 8%). Since any shift downward from the reference point (i.e., 6%) is perceived as a loss, people tend to display risk-seeking behavior to return to the status quo. Levy [27] (p. 93) explained that politicians tend to accommodate themselves to gains but take large risks to recover losses. Since a situation with increasing unemployment rates would constitute a domain of loss, many politicians would be inclined to vote for a digitalization policy with a high probability of failure (*p* > 0.30) in the hopes of recovering the losses.

However, risk preferences reverse in the face of a low probability of gains (*p* < 0.30), leading to risk-seeking in gains and risk-aversion in losses. Imagine that the economy is framed as positive due to lower levels of public debt from the previous year to the present year. According to the value function, politicians would accommodate themselves to the gain and become risk-averse toward a digitalization policy with a small chance of failure (e.g., increasing public spending to finance ICT infrastructure) and a large chance of success (e.g., reducing public spending even further due to higher public sector efficiency). However, given that small probabilities of large gains are overweighted, politicians would be inclined to take the risk and vote for the policy presented with a small chance of a large win (e.g., winning the next election in an unlikely scenario). Thus, instead of becoming risk-averse, politicians would become risk-seeking in the domain of gains, a situation that leads to the distinctive four-fold pattern of risk attitudes.

The economy is not the only relevant area politicians must consider when making decisions in digitalization policies. Some of the biggest challenges in digitalization are related to the security and the privacy of data. In situations of unlikely gains and unlikely losses, as in the case of data privacy, the application of the four-fold pattern becomes even more relevant to examine the risk preferences of politicians in digitalization.

## 4. Risk Preferences and the Data Privacy Dimension

Needless to say, the uncertainty created by digitalization is not exclusive to the economy. As technology continues to advance, so do issues related to data privacy [3]. Governments around the world are increasingly collecting, storing, and analyzing large amounts of data on citizens’ activities. Although more information allows governments to reduce costs and become more efficient in public service delivery, it increases, rather than decreases, the risk of data breaches and misuse [51]. Perhaps the best representation of the threats that digitalization may pose to data privacy is the Internet of Things (IoT). IoT systems connect multiple devices from a vast number of different sources, all interacting with each other [52]. This means that protecting against data breaches, therefore, becomes a security nightmare, as this interconnectivity of multiple devices offers a variety of vectors to malicious hackers. Even big technology companies, including Yahoo, Amazon, Google, and Facebook, have already been subject to innumerable data breaches [53]. In the case of the healthcare sector, the increasing gathering and sharing of individuals’ sensitive healthcare data have raised serious concerns regarding profiling, tracking, discrimination, exclusion, and state surveillance [53] (p. 251).

Not surprisingly, the healthcare sector has been one of the main areas affected by digitalization [54]. While the gathering, storing, and sharing of medical information have the potential to enhance efficiency in the healthcare sector by reducing healthcare costs, for instance, it also carries serious risks to the privacy of health data. This is why data privacy in the healthcare sector has been a matter of intense debate, particularly about data protection laws and regulations [55,56].

Much like the economy, data privacy is becoming a partisan issue, in which politicians across all political spectra are increasingly having to make choices concerning the issue of data privacy. For example, let us assume that the context of privacy and security of the personal healthcare data in a particular country is located in the domain of gains due to increased public sector investment aimed at securing sensitive health data (e.g., diagnoses, procedures, lab values, demographics, medication, provider) shared across public organizations and agencies. Now imagine that a digitalization policy has been proposed to limit the use of health data beyond its purposes. Let us assume that, for various reasons, the policy has an 80% chance of success (i.e., prevents public health providers from sharing health data beyond the purposes for which it was initially collected). Given that high probabilities are underweighted, it would be reasonable to assume that most politicians would lean toward risk-averse behavior. The domain of gain would induce politicians to abide by the current situation, or the status quo, rather than risk losing it in favor of a new alternative. The S-shaped subjective value function is concave in the domain of gains, which supports risk-aversion. The situation based on the outcome ‘increased public sector investment in data security’ sets the positive frame and hence the reference point. Anything below is considered a loss.

On the other hand, if the situation was framed as a high-risk security information environment where sensitive health data have been subjected to increasing hacking activity, politicians would be thrown into a domain of losses. In such a scenario, a digitalization policy described with an 80% chance of failure would motivate politicians to prefer the outcome with risk (i.e., probabilities) over the sure option, given that the underweighting of high probabilities reinforces the pattern of risk-seeking in losses.

However, risk preferences could shift directions. As predicted by the four-fold pattern, individuals tend to overweight small chances (i.e., *p* < 0.33) of unlikely large gains, which leads to risk-seeking instead of risk-aversion and to overweighting small changes of unlikely large losses, which leads to risk-aversion instead of risk-seeking [20]. In political decisions involving data privacy, the reverse of risk preferences would depend on the uniqueness of the situation. Consider the following: the current level of security of health data is satisfactory, with no major data breaches over the last year (domain of gain). Now, imagine that a digitalization policy designed to increase the level of data security by aggregating the personal healthcare data of all citizens into one single national database is proposed. The policy has only a 20% chance of success. Nevertheless, many politicians would be likely to vote for the policy (i.e., risk-seeking), even in the domain of gain, as the opportunity to aggregate citizens’ healthcare data into a national database for various purposes would be seen as an unlikely large gain too good to be wasted.

In contrast, a situation framed as negative because of increasing hacking activity (domain of loss) could induce risk-averse behavior from politicians due to a small probability of large losses. Following the previous scenario, imagine that a digitalization policy is proposed to reduce the risk of data hacking by implementing a centralized healthcare data platform system. The policy has a 20% chance of failure, that is, of increasing the risk of data hacking by exposing sensitive health data such as substance abuse, psychiatric care, and medication history of millions of citizens to a foreign enemy country, which could be used to identify, target, and recruit national informants [57]. This unlikely small probability of large losses would reverse the risk preference in the domain of losses by pressuring politicians to become risk-averse.

Nevertheless, outcomes in different dimensions can play against each other by shifting risk preferences depending on how each dimension is framed and how the outcomes’ probabilities are weighted.

## 5. Risk Preferences with Outcomes in Multiple Dimensions

Examining how politicians weigh the risks and the benefits of digital technologies when outcomes are multidimensional is very important for identifying—and even predicting—the risk preferences of politicians concerning digitalization policies.

How would politicians react when facing a gain in the economic dimension (e.g., higher employment numbers) but a loss in the data privacy dimension (e.g., lower levels of personal data privacy or government surveillance scandals)? Which dimension would dominate? It would depend on how each dimension is framed and how the outcomes’ probabilities in each dimension are weighed.

For some politicians, the economic benefits might outweigh the data privacy risks involved in the decision. For others, digitalization might not be seen as economically beneficial but as a threat to the data privacy of citizens. However, a higher risk of data breaches and data misuse might not be seen by other politicians as a potential threat if presented as essential in creating jobs, increasing wages, and enlarging municipal income tax revenues, particularly in a negative economic context. Thus, some politicians might consent to the loss of data ownership, thinking that there is no option but to sacrifice personal privacy for economic growth. Others might believe that fostering economic growth at the expense of a higher risk of data misuse is not worth pursuing, especially if politicians use a domain of loss in data privacy as the reference point.

The way a situation is framed determines how the individual interprets the reference point [58]. Thus, times of good economic performance, when things are going well, place politicians in a domain of gains, leading to risk-averse behavior. Likewise, a scenario in which there has been an increasing number of cyberattacks or misuses of data would place politicians in the domain of losses concerning data privacy, leading to risk-seeking behavior. In the former case, the reference point, or the status quo, is the ‘good economic performance’. Anything below it is considered a loss, and anything above it is considered a gain. Due to risk-aversion, people tend to choose certainty, or a sure option, over a probabilistic option with greater expected value. The underweighting of moderate and high probabilities relative to sure options contributes to risk aversion in the domain of gains by reducing the attractiveness of positive chance [58] (p. 145). Even if faced with a 50/50 percent chance of winning with the same absolute value, people would still prefer to prioritize the status quo by choosing the sure option [30].

Nevertheless, people tend to overweight small probabilities of unlikely large gains and large losses. They perceive a 5% chance of occurrence as far more likely than it is. For instance, imagine that there has been a considerable increase in hacking activities against health data (i.e., domain of loss) in a particular county over the last year. Amid this, a digitalization policy intended to reduce the risk of cyberattacks or misuse of sensitive health data is proposed. Instead of a single dimension, the policy contains multiple dimensions (i.e., economy and data privacy) with different probabilities of occurrence. Now imagine that the policy offers a small chance of failure (*p* < 0.10) in reducing the risk of hacking against health data. Suppose now that the economic dimension is located in the domain of gain with a lower unemployment rate in comparison to last year (e.g., from 5.5% to 4.5%) and that the policy offers a small chance of success (*p* > 0.20) in preventing the decrease in the number of jobs in the healthcare sector due to the automation of tasks.

As a result, a competition for framing and outcomes would take place to determine which one is dominant. According to the four-fold pattern, some politicians would value the economy over data privacy. They would choose to focus on the small chance of success in the economic dimension and vote for the policy (i.e., risk-seeking in the domain of gain). Others would take into consideration the small chance of failure in the data privacy dimension and vote against the policy (i.e., risk-aversion in the domain of loss).

In sum, the way people respond to gain/loss frames and the way they weigh probabilities contribute to their risk preferences. Recall that the value function is applied to a reference point that distinguishes between losses and gains [23]. In this particular case, because the loss frame in data privacy—in comparison to the gain frame in the economy—sets the perceived utility of the outcome in data privacy below the perceived utility of the outcome in the economy, politicians concerned with data privacy would be most likely to vote for the policy (i.e., risk-seeking). Data privacy would become the reference point; that is, it would be used to determine a domain of losses by inducing risk-seeking behavior. This pattern of behavior follows the shape of the value function (i.e., risk-seeking for losses but risk-aversion for gains). However, since people’s risk attitude is determined not only by the shape of the value function but also by the shape of the probability weighting function [20], small probabilities of gains and losses could reverse the risk preference and shift the reference point.

The multidimensional aspect of digitalization can become even more complex. Let us assume that the economy is in the domain of losses. Let us also say that the economic situation has been worsened by the data privacy dimension, as regulations intended to protect data from misuse have increased compliance costs, affecting jobs and investments in new and innovative firms. This has been the case in Europe, as the rollout of the General Data Protection Regulation (GDPR) is estimated to have cost European startups up to 39,000 jobs [59]. Now, let us assume that there has been increasing public concern over data privacy, intensified by incidents of data misuse and data fraud. Inevitably, this would throw politicians into the domain of loss in the data privacy dimension. Given that both dimensions are in the domain of loss, the status quo in both cases would be perceived as unacceptable by politicians. 

Now let us assume that a digitalization policy is proposed. Given that the situation in each dimension is already located in the domain of loss, one would assume risk-seeking behavior in either dimension, as it is accounted for by the value function. However, risk preferences are also determined by the probability weighting function. Let us say that the proposed policy has a large chance (*p* = 0.80) of failure in the economy dimension (e.g., destroying jobs due to automation) and a small chance (*p* = 0.20) of failure in the data privacy dimension (e.g., exposing the healthcare data of citizens to foreign hackers). Given that people underweight the likelihood of high probability events, the economic dimension would induce risk-seeking behavior. On the other hand, a small chance of unlikely large losses in the data privacy dimension would pull politicians toward risk-aversion, as small chances of losing are overweighted. In both cases, the outcomes are located below the reference point, which can be considered a precrisis status quo, constituting a domain of loss. Given that people tend to overreact to small probabilities, but underreact to large probabilities, one could assume that politicians would most likely place a higher weigh on the perceived utility value of data privacy than on the perceived utility value of economy. They would choose the data privacy dimension as the reference point and therefore display a risk-averse behavior. Overall, predicting the risk preferences of politicians is not an easy task. The different dimensions and their interrelations, the way issues are framed, and the probability of each outcome all influence the risk preferences of politicians.

## 6. Discussion

Properly applying prospect theory to examine politicians’ decisions can expand our understanding of the conditions under which politicians make decisions in highly complex policy environments. Digitalization has been a disruptive force across governments, economies, and daily life activities. It is a complex and dynamic phenomenon with decisions dominated by high levels of risk due to the uncertainty of outcomes across multiple areas. Public officials responsible for approving laws and regulations might have a hard time making decisions about digitalization, as they have to consider trade-offs between critical areas.

Under important assumptions—value function and probability weighting function—prospect theory offers us the ideal theoretical lens to access the conditions influencing the risk preferences of politicians in these complex policy environments. Digitalization, particularly in the healthcare sector, offers us the ideal contextual case to describe how politicians might behave in the face of outcomes with multiple dimensions. I have argued that the way the situation is framed affects the risk preferences of politicians in digitalization. For instance, describing the economy in a state of loss is likely to incite risk-seeking behavior, leading politicians to be more welcoming of digitalization policies with higher risks of failure. Likewise, framing the economy as positive is likely to lead to risk-averse behavior. As people make decisions with respect to a reference point, outcomes below the reference point are seen as losses, prompting risk-seeking behavior, and outcomes above the reference point are perceived as gains, leading to risk-averse behavior. Manipulating the perception of a situation—e.g., economic indicators such as employment—by means of framing—has the potential to affect politicians’ risk preferences. Empirical research indicates that politicians are susceptible to cognitive bias [14,15,16,17,18]; therefore, it becomes very important to understand how framing can affect politicians’ risk preferences in digitalization, since poorly designed and highly negative policies may be approved as a result of framing effects.

Another important aspect of decision-making under risk is probability weighting. According to prospect theory, people tend not to treat probability linearly. Instead, they are inclined to overweight small probabilities and underweight large probabilities. I have attempted to illustrate that probability weighting is a key feature in the theoretical applications of prospect theory to political decisions in digitalization, which may reverse the trajectory of risk preferences. This shows that attempting to describe or predict the risk preferences of politicians based solely on the value function is not ideal since the way people weigh probabilities plays an essential role in risk behavior.

Last, I have discussed how the combination of the value function and the probability weighting function might help us to understand the risk behavior of politicians in decisions involving multiple dimensions. Policies in digitalization usually involve trade-offs between multiple outcomes (e.g., a loss in one area vs. a gain in another) that depend on how the situation is framed and how each dimension is weighted. Some politicians might value the economy over data privacy, while for others, the exact opposite may be true. These trade-offs are challenging to make since it is quite difficult to pinpoint the consequences of a decision. In addition, politicians are constantly worried about the electoral consequences of their decisions [60] (p. 22), with empirical research showing that politicians are inclined toward choices in which they are held accountable (see [15]). Thus, the high level of uncertainty in digitalization policies might drive politicians to choose policies that are electorally safe for one segment of society but detrimental to another. Framing effects and people’s tendency to judge probability unliterally may further contribute to politicians making poor policy choices.

## 7. Conclusions

We still lack a comprehensive understanding of the cognitive processes underlying the choices made by politicians. Given the increasing complexity of issues in many countries around the world, especially those involving the digitalization of economies and societies, it becomes paramount to study the conditions influencing the risk preferences of politicians. This paper offers the first systematic theoretical examination of the possible conditions affecting the risk preference of politicians in a complex policy setting such as digitalization. Although limitations apply to theoretical analysis, having a first theoretical-based discussion can provide the basis for future empirical studies. It invites scholars and practitioners in the fields of political science, political psychology, public policy, and public administration, among others, to design and implement empirical studies to measure the risk preferences of politicians when the outcomes of their decisions are unknown or hard to predict, which is usually the case in policies involving the digitalization of crucial aspects of governments, businesses, and daily life. Their results could provide valuable insights into how politicians behave in highly complex and critical policy contexts.

## Data Availability

Not applicable.
